# LGBTIQ+ Homelessness: A Review of the Literature

**DOI:** 10.3390/ijerph16152677

**Published:** 2019-07-26

**Authors:** Brodie Fraser, Nevil Pierse, Elinor Chisholm, Hera Cook

**Affiliations:** He Kainga Oranga/Housing and Health Research Group, University of Otago Wellington, Wellington 6242, New Zealand

**Keywords:** homelessness, LGBT, intersectionality, LGBTIQ+, discrimination, housing, exclusion, queer

## Abstract

Lesbian, Gay, Bisexual, Transgender, Intersex, and Queer (LGBTIQ+) people’s experiences of homelessness is an under-explored area of housing and homelessness studies, despite this group making up 20–40% of homeless populations. Despite this, much of the existing literature focuses on specific elements of LGBTIQ+ homelessness, and often does not consider the intersections of these elements, instead placing them into individual siloes. Our approach is an intersectional one; this paper identifies the key themes in the existing research, and analyses how these themes interact to reinforce the discrimination and stigma faced by LGBTIQ+ people who experience homelessness. This intersectional-systems thinking approach to LGBTIQ+ homelessness can be used to develop well-informed, culturally sensitive support programmes.

## 1. Introduction

The aim of this review is to explore the intersections of factors associated with both homelessness and Lesbian, Gay, Bisexual, Transgender, Intersex, Queer, and other diverse sexual orientations and gender identities (LGBTIQ+) in order to examine their role in experiences of LGBTIQ+ homelessness. Multiple studies have shown LGBTIQ+ people are more likely to be homeless than non-LGBTIQ+ people [1,2,3,4]. LGBTIQ+ people comprise an estimated 20–40% of homeless populations, whilst only comprising 5–10% of the wider population [5,6]. Despite this, much of the existing literature focuses on specific elements of LGBTIQ+ homelessness, and often does not consider the intersections of these elements. In researching factors of LGBTIQ+ homelessness in siloes, we are at risk of falling into scattered, disjointed understandings of the issue, resulting in piecemeal solutions [6,7]. Intersectional research conversations need to occur. The goal of this review is to identify the key themes in the existing research, and analyse how these themes interact to reinforce the discrimination and stigma faced by LGBTIQ+ people who experience homelessness.

Edgar [8] defines homelessness as exclusion from physical, social, and legal domains, and exclusion from any one or two of these domains is defined as housing exclusion. Amore et al. [9] argue homelessness should be replaced by the concept of severe housing deprivation, which includes two main criteria; (1) that a person is living in severely inadequate housing due to (2) a lack of access to housing that meets a minimum adequacy standard (rather than living in such circumstances by choice) [9]. Severe housing deprivation consists of experiencing any two of three categories; inadequate privacy and control; inadequate security of tenure; and inadequate/uninhabitable structure [9]. Homelessness thus includes rough sleeping, couch surfing, living in shelters and women’s refuges, and living in cars, caravans, and tents [9]. The Amore et al. definition is used in this paper, as it is a more comprehensive definition of homelessness.

This literature review aims to clarify the relationships between LGBTIQ+ identity and the key themes in the literature. The key themes are; poverty, ethnicity and racism, substance use, mental health, sexual abuse, foster care, LGBTIQ+ discrimination and stigma, family, survival sex and sex work, physical ill-health and Human Immunodeficiency Virus (HIV), and shelter inaccessibility. This review focuses on the interconnections between these experiences, using Kimberlé Crenshaw’s [10] theory of intersectionality, which argues people experience different and multiple oppressions in response to their different identities—for example, a person living in poverty, a person’s ethnicity, their gender—which compounds negative health and social outcomes. The concept of intersectionality highlights the interactions between people’s multiples identities and systems of oppression and the resulting complex outcomes [11]. This paper examines the interactions between multiple identities, such as LGBTIQ+, ethnicity, and systemic failures such as discrimination and stigma, in order to explore experiences of LGBTIQ+ homelessness. It is necessary to understand these identities and experiences together, as the way in which people identify is not based solely on one category, but rather, is a collection of multiple identities and experiences [12]. Intersectionality encourages us to consider how upstream social determinants (such as racism, sexism, classism, transphobia, and queerphobia) form interlocking systems of oppression which shape the experience of people with multi-dimensional identities [13].

## 2. Methods

Literature searches were performed using PubMed, ProQuest, ScienceDirect, Scopus, MedLine, and Google Scholar. Four searches were run in August 2017 and again in August 2018. These were; “LGBT Homelessness;” “Queer Homelessness;” “LGBT Housing First;” and “Queer Housing First.” Most results came from the searches containing ‘homelessness’ as a key term, with searches including ‘Housing First’ adding a few more results. It was decided to use LGBT, instead of LGBTIQ+, in the searches as LGBT is the more commonly used acronym, and thus more likely to return searches. The searches returned a total of one hundred articles with relevant keywords. The article abstracts were reviewed for relevance, which was determined by coverage of LGBTIQ+ identity and homelessness; a total of 27 articles were used in this review. An additional 26 articles were found via their reference lists. Due to the limited amount of relevant research, it was decided not to have a specific start date for articles. Each article was coded for the key themes it covered; this created a “literature map” that enabled a visualisation of the 12 most prominent, or key, themes and their prevalence [14].

## 3. Results

### 3.1. Key Themes

**Figure 1:**
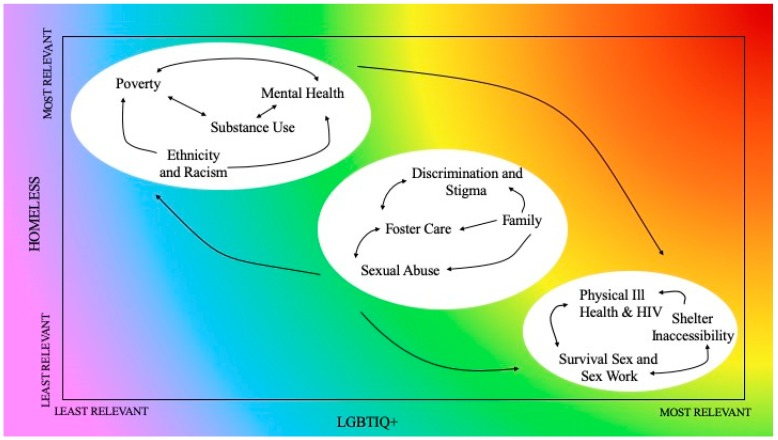
Intersections of LGBTIQ+ Identity and Experiences of Homelessness. The groupings from left to right are; proximate causes of homelessness, systems failures in early life, and experiences during homelessness.

The key themes in the literature were; poverty, ethnicity and racism, substance use, mental health, sexual abuse, foster care, LGBTIQ+ discrimination and stigma, family, survival sex and sex work, physical ill-health and HIV, and shelter inaccessibility. The relationships between them are displayed in Figure 1:

Figure 1 displays the relevance of each theme to LGBTIQ+ identity along the horizontal axis, and homelessness along the vertical axis. The themes are positioned in order of least relevant to most relevant; themes closest to the inner bottom left-hand corner are least relevant, and those closest to the outer edges are more relevant. The arrows between the themes and groupings indicate their relationships. Single-direction arrows indicate a one-way relationship, and multi-direction arrows indicate a two-way relationship. The groupings were produced through examination of the literature to determine how strongly each was linked, firstly to homelessness, and secondly to LGBTIQ+ identity. The first grouping is proximate causes of homelessness, including poverty, ethnicity and racism, substance use, and mental health. The second grouping is failures of support systems in early life, including sexual abuse, foster care, discrimination and stigma, and family. The third grouping is experiences during homelessness, including survival sex and sex work, physical ill-health and HIV, and shelter inaccessibility. Figure 1 shows the relationships between the themes that act to produce LGBTIQ+ homelessness. Analysing these relationships produces a holistic conceptualisation of LGBTIQ+ homelessness. In the following analysis, the themes are discussed in the order in which they fall within their groupings.

### 3.2. Proximate Causes of Homelessness

#### 3.2.1. Poverty

Poverty is the main driver of homelessness; it is ubiquitous among people who experience homelessness and escaping poverty becomes more difficult when people are homeless [15,16,17]. Poverty and economic instability create a state of precarity, which can lead to a number of difficulties such as a difficulty maintaining housing, poor mental health, and addiction [16]. Thus, the relationship between poverty and homelessness is a complex one; it moves in multiple directions and is intimately connected to the aforementioned factors [18]. High levels of income inequality and low levels of social welfare are associated with increased rates of homelessness [16]. Poverty is a structural factor that is intimately linked to homelessness [16,18]. There is, thus, a strong link between poverty and experiences of homelessness. There is a weaker link between poverty and LGBTIQ+ identities. This relationship is nuanced; different demographics within the wider LGBTIQ+ community have varying experiences of poverty; white lesbian women are likely to be high earners, while white gay men and gay and lesbian people of colour are more likely to be lower earners [19,20]. Poverty is also connected to the other issues within the proximate causes of homelessness grouping; it is both a driver and a consequence of addiction and/or mental health issues [16]. Poverty directly influences how people experience homelessness.

#### 3.2.2. Ethnicity and Racism

Racism, systematic inequality, and historical trauma mean that ethnic/racial minorities are more likely to experience homelessness in comparison to dominant ethnic groups. In the USA, for example, roughly 42% of people who are homeless are African American, and roughly 20% are Hispanic, despite each group compromising just over 12% percent of the total population [12]. This over-representation occurs due to a myriad of complex factors such as social exclusion in the areas of wealth, income, housing, and imprisonment [16]. For LGBTIQ+ ethnic minorities, the intersection of minority identities increases the odds of adverse experiences through the greater likelihood they will also suffer poverty, discrimination, and victimisation. Page [12] argues this intersectionality gives homeless LGBTIQ+ people of colour disproportionately high chances of experiencing hardship and emotional distress. Multiple intersecting identities can result in a host of negative health and social outcomes [12,13]. Exploring these intersections is necessary to fully understand the experiences and needs of ethnic--minority LGBTIQ+ homeless people as the discrimination racial and sexual minorities face is intensified when they are homeless; homelessness becomes an added stressor [12].

#### 3.2.3. Substance Use

Substance abuse is a proximate cause of homelessness, and can be exacerbated once people become homeless [21]. People who are homeless use drugs and alcohol at far greater rates than the wider population, with studies finding 40–70% of people who are homeless reporting alcohol and drug dependence [21,22,23,24]. Substance use is a broad issue across all demographics of homeless populations [24]. However, LGBTIQ+ homeless people have higher rates of substance use when compared to non-LGBTIQ+ homeless people [2,25,26,27,28]. In addition to this, transgender homeless people have even higher substance use rates than Lesbian, Gay, and Bisexual (LGB) homeless people [28]. Both Gattis [2] and Van Leeuwen et al. [25] found homeless LGBTIQ+ people were more likely than homeless non-LGBTIQ+ people to have reported usage of 20 out of 21 illicit substances. Whitbeck et al. [29] found lesbian females were more likely than heterosexual females (61.4% versus 35.5%) to meet criteria for alcohol abuse. Flentje et al. [1] reported similar findings amongst homeless populations: LGBTIQ+ males had lower rates of drug or alcohol abuse in comparison to heterosexual males, whilst LGBTIQ+ women had 6.33 times the odds of drug and alcohol abuse in comparison to heterosexual women. These studies indicate the relationship between substance use and homelessness is stronger than that between substance use and LGBTIQ+ identity in and of itself; LGBTIQ+ identity is an added factor that intersects in a complex way with the other two.

#### 3.2.4. Mental Health

The relationship between homelessness and mental illness is bi-directional; homelessness can directly undermine mental health, and mental illness can directly lead to becoming homeless [30]. Homeless populations have high rates of mental illness, with studies finding between 42–80% of people who are homeless experience mental health struggles [24,31,32]. Non-homeless LGBTIQ+ populations experience mental health issues at somewhat lower rates of about 40% [33]. This indicates poor mental health is an enormous issue for homeless populations, and a significant issue for the LGBTIQ+ population. Whitbeck et al. [29] compared LGB homeless youth to heterosexual homeless youth on levels of mental disorders such as major depressive episodes and post-traumatic stress disorder (PTSD), suicidal ideation, and suicide attempts. Overall, LGB participants had significantly higher rates of depression (41.3% versus 28.5%), PTSD (47.6% versus 33.4%), suicidal ideation (73.0% versus 53.2%), and suicide attempts (57.1% versus 33.7%). Noell and Ochs [34] also found LGB youth had higher rates of depression, suicidal ideation, and suicide attempts in comparison to their heterosexual counterparts. LGBTIQ+ people who are homeless thus face greater long-term mental health issues. This may result from prior experiences of strained family relationships, high levels of sexual and physical abuse, and can be reinforced by intersecting identities. Flentje et al. [1] found cisgender Gay, Bisexual, and Queer men were 2.68 times as likely to have a psychiatric condition and 3.47 times as likely to have PTSD compared to their heterosexual counterparts. Amongst homeless cisgender women, those who identified as LGBQ had 5.16 times the odds of a psychiatric condition in comparison to homeless heterosexual cisgender women [1]. Transgender men were 3.78 times as likely to have a psychiatric condition and 3.92 times as likely to have PTSD in comparison to cisgender men [1]. In comparison to cisgender women, transgender women had 3.31 times the odds of PTSD [1]. The experiences of homelessness and poor mental health combine with LGBTIQ+ identity to deepen the systems failure for LGBTIQ+ people who are homeless.

### 3.3. System Failures in Early Life

#### 3.3.1. Sexual Abuse

Sexual abuse is both a driver to, and consequence of, homelessness. People who are homeless experience higher levels of sexual abuse (including rape, sexual assault, and sexual victimisation) than the wider population both before and during episodes of homelessness [35,36,37]. This relationship is even stronger among people with LGBTIQ+ identities [37,38,39]. Compared to non-LGBTIQ+ homeless people, LGBTIQ+-identifying homeless people face higher levels of sexual assault—particularly youth and transgender/gender diverse people [23,25,28,29,40,41,42,43,44]. Cray et al. [44] found homeless LGBTIQ+ youth had been sexually assaulted at three times the rate of non-LGBTIQ+ homeless youth. Whitbeck et al. [29] found 44.3% of LGB adolescents reported sexual abuse by an adult caretaker, in comparison to 22.3% of non-LGB adolescents. Furthermore, 58.7% of LGB homeless youth reported sexual victimisation on the streets, compared to 33.4% of heterosexual youth [29]. It is clear LGBTIQ+ homeless youth face disproportionately high rates of sexual abuse and sexual victimisation. Sexual abuse is connected to other themes in this review; notably, family, foster care, drug use, and mental health. Sexual abuse is a key reason as to why young people run away from home and become homeless [15,35,38,45]. Children and young people in foster care are susceptible to sexual abuse, often resulting in them running away from such environments [46,47]. Links have been found between childhood sexual abuse and substance use [48,49]. Sexual abuse also has a significant impact on mental health and wellbeing [29]. Sexual abuse is thus an example of how the key themes of this review intersect with each other, creating poor outcomes for LGBTIQ+ people who experience homelessness.

#### 3.3.2. Foster Care

Family breakdowns and unsafe family environments are major drivers to young people entering the foster care system. Being in foster care, and the instability associated with it, is a driver to becoming homeless: disproportionately high levels of young homeless people have been in foster care [3,7,45,50,51,52]. Due to higher levels of family breakdown, those with LGBTIQ+ identities are over-represented in foster care systems [53,54,55,56,57,58]. LGBTIQ+ youth in foster care experience unique risks to achieving permanency and wellbeing such as rejection by foster families and lack of competency by caregivers and social workers [53,59]. Wilson and Kastanis [53] found 19.1% of the foster care population they surveyed identified as LGBTIQ+. This suggests LGBTIQ+ youth are more likely to have been in situations where it is safer for them to be placed into care. Furthermore, LGBTIQ+ foster youth were more likely than non-LGBTIQ+ youth to have been homeless at one point [53]. Feinstein et al. [60] found 56% of LGBTIQ+ foster youth had spent time sleeping rough because they felt safer on the streets than in their foster homes. Placing youth in foster care is intended to remove them from unsafe situations; it is worrying that such high levels of LGBTIQ+ youth do not feel safe in their foster homes. Clements and Rosenwald looked at foster parents’ perspectives on LGBTIQ+ youth in the foster system. They found four common themes about LGBTIQ+ youth in care; misconceptions regarding LGBTIQ+ identity, fears of LGBTIQ+ children molesting their own children, differences in attitudes towards children of differing LGBTIQ+ identities, and anti-LGBTIQ+ religious beliefs [61]. The intersection of foster care and LGBTIQ+ identity place these youths at greater risk of experiencing homelessness than non-foster and non-LGBTIQ+ youth.

#### 3.3.3. LGBTIQ+ Discrimination and Stigma

Everyone has a right to be free from discrimination and valued for who they are; homeless LGBTIQ+ people face a huge barrier to realising this. While the global movement for LGBTIQ+ equality is growing and achieving significant gains, the LGBTIQ+ community still faces discrimination and stigma. Kidd [36] found a relationship between LGBTIQ+ identity and the amount of guilt, shame, and self-blame directly related to levels of stigma reported, which influences mental and emotional wellbeing. This can stem from issues such as family- and/or foster-care-related conflicts—often the first LGBTIQ+-related discrimination people face is from within their families. Discrimination based on homelessness then compounds this, which could result in greater levels of self-blame amongst LGBTIQ+ people who are homeless. High levels of self-blame suggest homeless LGBTIQ+ youth are internalising stigma, which is a central aspect of how discrimination affects mental health [36]. LGBTIQ+ homeless people face discrimination regarding their LGBTIQ+ identity, and their homelessness status [2]. These factors can intersect with other factors such as ethnicity, mental illness, and disability. Gattis [2] found LGBTIQ+ respondents faced higher levels of stigma related to being homeless compared to non-LGBTIQ+ respondents. He also reported homeless LGBTIQ+ youth had experienced more discrimination in the past year when compared to homeless non-LGBTIQ+ youth [2]. LGBTIQ+ homeless people experience greater levels of discrimination and stigma than non-LGBTIQ+ homeless people, both on the basis of homeless status and LGBTIQ+ identity. Stigma can lead to feelings of isolation, loneliness, feeling trapped, and low self-esteem. This can, in turn, make it difficult for people to escape homelessness due to the negative effects of stigma and discrimination.

#### 3.3.4. Family

The breakdown of family relationships is an important risk factor for homelessness. This is especially so for LGBTIQ+ people; it is the main driver of homelessness for LGBTIQ+ youth [5,41,55,62,63,64,65,66,67]. Castellanos [62] reported three main pathways into homelessness amongst LGBTIQ+ youth. The first was disclosure of LGBTIQ+ identity exacerbating existing family conflicts, resulting in the young person being kicked out of the home, or choosing to leave [62]. The second was that youth left home, or were forced to leave, over their LGBTIQ+ identity [62]. The third emerged where young people had been released from state supervision back into the care of their family, and family conflict became intolerable due to disclosure of their LGBTIQ+ identity [62]. All three pathways are linked to negative family responses of the young person’s LGBTIQ+ identity. Shelton explains that for some youth, episodes of homelessness saved their lives due to the discrimination they experienced within their family homes; several transgender and gender-expansive youth stated they would likely have committed suicide if they had not left their families and become homeless [68]. Abramovich [65] found the most common cause of LGBTIQ+ youth becoming homeless was identity-based family conflict that arose as a result of these youth coming out. Thus, it is important service provision does not rely on young people reconnecting with their families; for many, improving family relationships and communication is not possible due to the discrimination they face from their families. Negative family attitudes towards LGBTIQ+ identity are a strong driver of homelessness.

### 3.4. Experiences During Homelessness

#### 3.4.1. Survival Sex and Sex Work

Poverty and homelessness create a lack of options that may lead to survival sex and sex work. Survival sex is defined as trading sex to meet one’s survival needs [5,29,69]. Survival sex is often a non-cash exchange, rather than a more straightforward transaction, that is a response to poverty, and may result in economic dependence (instead of a professional transaction), the term survival sex is used [69]. LGBTIQ+ homeless populations engage in riskier behaviours and survival strategies while on the street when compared to their non-LGBTIQ+ counterparts [29,70]. Existing literature indicates LGBTIQ+ people who are homeless engage in survival sex and sex work at consistently higher rates than non-LGBTIQ+ people who are homeless [31,34,43,69,71]. Childhood sexual abuse and entry into sex work have been linked by Lankenau et al. [26]: previously abused youth know there exists a demand for certain types of sexual activity. Ream et al. [57] found LGBTIQ+ youth were very aware of the risks associated with survival sex and sex work. Marshall et al. [43] found in comparison to their heterosexual peers, homeless sexual minority youth who engaged in survival sex reported significantly higher numbers of clients, as well as inconsistent condom use with clients, putting them at greater risk of contracting sexually transmitted infections. Survival sex and sex work is thus a common experience for LGBTIQ+ people who are homeless.

#### 3.4.2. Physical Ill-Health and Human Immunodeficiency Virus

Physical ill-health is a concerning issue amongst homeless populations. Despite homeless people’s many vulnerabilities to poor health—such as injuries, harsh weather exposure, and assault —there are numerous barriers to care—such as cost, lack of transport, and fear of judgement [30]. This suggests homeless people can be reluctant to use health services and delay seeking help until their conditions deteriorate, increasing the risk of hospitalisation [30]. Despite this, there is limited information on the experiences of those who identify as LGBTIQ+. Chang et al. [30] looked at hospitalisation rates amongst homeless youth who used drugs; 75.9% of the respondents who had been hospitalised in the past six months identified as LGBTIQ+. Gattis [2] found LGBTIQ+ homeless youth were significantly more likely to be the victims of physical assault than heterosexual homeless youths. This suggests the disproportionate levels of discrimination LGBTIQ+ homeless people face is resulting in higher levels of physical assault [2]. A specific physical health issue both LGBTQ+ and homeless populations face is HIV, and interestingly, the literature did not focus on other Sexually Transmitted Diseases, despite them being serious diseases. LGBTIQ+ homeless people have a disproportionately high rate of HIV infection when compared to the non-LGBTIQ+ homeless population [71,72]. However, the literature also indicated LGBTIQ+ people who are homeless are more likely to have recently been tested for HIV than non-LGBTIQ+ people who are homeless [73]. Improved public education and awareness, targeted at both homeless and wider populations, as well as accessibility of testing, has increased the levels of testing [72,74]. Greater levels of HIV testing can result in earlier detection and safer sexual practices [73]. Thus, homeless populations are vulnerable to physical ill-health, and LGBTIQ+ homeless populations are particularly vulnerable to HIV infection, despite their high rates of testing.

#### 3.4.3. Shelter Inaccessibility

Shelters are intended to be a place of support and refuge for people experiencing homelessness, however, for LGBTIQ+-identifying people they can be a site of vulnerability and danger. Despite the overrepresentation of LGBTIQ+ people in homeless populations, service providers are often under-prepared to work with LGBTIQ+ homeless people [75]. Maccio and Ferguson [75] argue the result of this is a dearth of services meeting the needs of LGBTIQ+ people, such as private showers and LGBTIQ+ sensitivity training for staff, and the available supply of services are alienating to LGBTIQ+ clients due to their heteronormative and cis-normative bias. Abramovich [76] found LGBTIQ+ people going into shelters are fearful due to the danger resulting from discrimination that they are likely to face. The lack of training and understanding from staff can result in staff being queerphobic, and/or not prioritising intervening in incidents of queerphobia [76]. Transgender and gender-diverse people are often denied access to shelters due to their gender identity, particularly in single-gender shelters that lack policy regarding gender diversity [76,77,78]. They have historically been excluded from single-gender shelters, which leaves them vulnerable to violence, murder, and other safety risks [79]. When transgender and gender-diverse people are admitted into shelters and assigned placement based on their anatomic sex, they are vulnerable to aggression and sexual assault [79]. In models used in addressing homelessness, shelters are often the first step in accessing support; new models such as Housing First have the potential to work better for LGBTIQ+ people as secure housing is the starting point in addressing homelessness, instead of the end point [80].

## 4. Discussion

This paper builds on existing LGBTIQ+ homeless literature, examining the intersections of key themes faced by both people who identify as LGBTIQ+ and people who are homeless. We propose a new way of categorising and visualising the key themes, as presented in Figure 1. This diagram places the themes in relation to each other, thus enabling them to be understood synergistically.

The first grouping was of proximate causes of homelessness and included the poverty, ethnicity and racism, substance use, and mental health themes. The racism that ethnic minorities face can directly contribute to poverty and poor mental health, which can, in turn, lead to a person becoming homeless [12]. Similarly, poverty can lead to poor mental health and substance use, just as poor mental health and substance use can lead to poverty [16,81]. Thus, the relationship between all these factors is bi-directional; each can lead to the other. These amplifications of multiple negative factors that cause homelessness show a clear failure to care for those who are most vulnerable.

The second grouping was of systematic failures and included sexual abuse, foster care, discrimination and stigma, and family. LGBTIQ+ identity has a considerable role within this grouping and its relationship to these themes acts as longer-term drivers of homelessness. As shown in the results, unsafe family situations can result in foster care placement. Foster care has a bi-directional relationship with sexual abuse. The literature showed that sexual abuse (particularly within family structures) can result in a young person being placed into foster care [29]. Youth might then experience sexual abuse within the foster care system [82]. Foster care has a bi-directional relationship with discrimination and stigma; young people might experience high levels of discrimination and thus be placed into foster care; where they might experience further, or initial, discrimination and stigma due to their LGBTIQ+ identity [53,59,82,83]. Failures in care systems have the potential to induce substance abuse and poor mental health. Additionally, they can produce economic and social vulnerability which encourages people to engage in survival sex and sex work [59]. Survival sex may also enable people to provide for themselves in order for them to be able to leave untenable family or foster care situations. Mental health is affected by all of the themes in this grouping; experiencing any of these four systematic failures can result in poor mental health [2,29,59,68]. Thus, interventions targeted at addressing these factors must also consider the ways in which they impact on people’s mental wellbeing, and ensure the intersectional nature of these issues is considered. It is primarily as a result of failures in these systems that LGBTIQ+ people experience poor mental health. The overlapping nature of these systematic failures shows a need for an inclusive, intersectional system to prevent homelessness.

The third grouping was of experiences during homelessness and included survival sex and sex work, physical ill-health and HIV, and shelter inaccessibility. This grouping has a strong immediate relationship to LGBTIQ+ identity, which indicates both direct and indirect discrimination in the ways in which the right to housing is realised. Survival sex and sex work have a bi-directional relationship with physical ill-health and HIV. Survival sex and sex work puts people at greater risk of contracting HIV and experiencing ill health [57]. Physical victimisation and ill health can result in people engaging in survival sex and sex work in order to meet their survival needs [84]. Survival sex and sex work also have a bi-directional relationship with shelter inaccessibility. When shelters are inaccessible to LGBTIQ+ people, they are more likely to engage in survival sex in order to find accommodation and/or money to meet their needs [84]. On the other side of this relationship, shelters have the potential to become inaccessible to people who are engaging in survival sex or sex work due to strict shelter policy regarding illegal behaviours. Both the proximate causes of homelessness and systematic failures lead to these experiences LGBTIQ+ people have while homeless.

Focusing primarily on negative aspects of LGBTIQ+ homelessness provides an incomplete, one-dimensional understanding of the issue [68]. This undermines the resourcefulness of these people and the instances of affirmation, connection, growth, and self-sufficiency they experience [68]. Reframing understandings of LGBTIQ+ homelessness has the ability to prevent us from confining interventions to risk-reduction models, and instead move towards strength-based models [68]. The relationships between the key themes indicates LGBTIQ+ people are willing to risk their health, and safety, in order to meet some of their needs, such as a place to sleep, food, money, and drugs. This suggests LGBTIQ+ people are often brave and resourceful in their engagement in behaviours that enable them to maintain their identity and to meet their survival needs. For example, this bravery can be seen when LGBTIQ+ people leave family and/or foster care situations in order to look after their physical and mental wellbeing. In viewing the survival strategies of LGBTIQ+ people as bravery and a lack of timidity, we are able to move from a purely deficit-focused view, to one that acknowledges their resiliency. Further research is needed to continue to expand upon this strengths-based understanding of LGBTIQ+ homelessness.

Literature from Hunter [85], Shelton [86], Abramovich [76], and Maccio and Ferguson [75,87] indicated a need for service providers to be aware of the particular issues LGBTIQ+ homeless people face, and work towards addressing these concerns. Hunter [85] identifies four main changes service providers should implement: private showering facilities, low-occupancy limits, housing programs that are prevented from discriminating on the basis of sexual orientation and/or gender expression, and specific LGBTIQ+ non-discrimination and sensitivity training for all staff. Ensign and Bell [88] found street-based people are more likely to visit emergency departments than shelter-based people. Thus, those in the shelter system have access to greater social and cultural capital than rough sleepers and other homeless people outside of the shelter system. It is possible LGBTIQ+ people are missing out on the support and knowledge provided by shelters, due to their often adverse feelings towards, and experiences of, shelters. With LGBTIQ+ people so over-represented in homeless populations, shelter staff need to undergo cultural competency training in order to provide culturally sensitive support [75].

As shown in Section 3.3, systems failures in early life are a key driver of LGBTIQ+ homelessness, particularly in early life. The interconnected nature of these system failures means we cannot address the failures in one system without addressing the failures in the others. The intersectionality of LGBTIQ+ people’s experiences with homelessness, poverty, and ethnicity makes them particularly vulnerable to these systems failures. This indicates a need to rethink and redesign these early-life systems, with targeted interventions for LGBTIQ+ people kept at the core of any changes. Such changes could include LGBTIQ+-specific protections in government policy, and LGBTIQ+ cultural competency training for those who work in related fields. Failing to address these needs breaches one’s right to housing and results in unequal outcomes, as evidenced by the high rates of LGBTIQ+ homelessness. This represents a failure in both policy and social wellbeing to support our most vulnerable.

The main limitation to this paper is related to the literature search; namely, that the initial search terms were somewhat inefficient at finding relevant research. As discussed in the Methods section, 27 relevant articles were obtained from the literature searches, and a further 26 came from reviewing the reference lists of those articles. Thus, almost half of the relevant articles did not come from the literature searches. Search terms could have thus been broadened and altered to capture a larger proportion of the relevant articles. It is possible that we have missed articles which use alternate terms and keywords such as “sexuality” or “sexual orientation” instead of “LGBT” and “Queer”, as we have used, which have only been rising in usage in recent decades. Another limitation of this study is the narrow geographic range of the literature; most articles come from North America, with a few from Australia and Europe. Further research would benefit from exploring the issue of LGBTIQ+ homelessness in a wider range of locations. This would broaden our understanding of the issue and allow us to see how LGBTIQ+ people’s experiences of homelessness differ across a range of social, political, cultural, and economic contexts.

## 5. Conclusions

People who identify as LGBTIQ+ experience homelessness at far greater levels than their non-LGBTIQ+ counterparts. This paper outlines the relationships between factors relating to LGBTIQ+ homelessness and proposes a systems-thinking approach through which to view them. We place these themes into three main groupings; proximate causes of homelessness, systems failures in early life, and experiences during homelessness. This systems-thinking approach to LGBTIQ+ homelessness can be used to develop well-informed, culturally sensitive support programmes, particularly in relation to early life intervention in order to prevent systems failures. Despite the increase in academic scholarship on the issue, more research is needed.
